# Prevalence of stroke in three semi-urban communities in middle-belt region of Nigeria: a door to door survey

**DOI:** 10.11604/pamj.2015.20.33.4594

**Published:** 2015-01-13

**Authors:** Emmanuel Olatunde Sanya, Olufemi Olumuyiwa Desalu, Feyiyemi Adepoju, Sunday Adedeji Aderibigbe, Akeem Shittu, Olabode Olaosebikan

**Affiliations:** 1 Departments of Medicine, University of Ilorin Teaching Hospital, Nigeria; 2 Departments of Ophthalmology, University of Ilorin Teaching Hospital, Nigeria; 3 Departments of Epidemiology and Community Health, University of Ilorin Teaching Hospital, Nigeria; 4 Departments of Hematology, University of Ilorin Teaching Hospital, Nigeria; 5 Departments of Chemical Pathology, University of Ilorin Teaching Hospital, Nigeria

**Keywords:** Stroke, crude-prevalence, semi-urban communities, epidemiology, Nigeria

## Abstract

**Introduction:**

The burden of stroke has been projected to increase for developing countries, but data are limited, especially in sub-Saharan Africa. This necessitated this study to determine the stroke prevalence in a semi urban community in middle-belt region of Nigeria.

**Methods:**

A two-phase door-to-door study was performed in three semi-urban communities of Kwara state; in the first phase 12,992 residents were screened and probable stroke cases were identified by trained health care workers. In the second phase individuals adjudged to be positive for stroke were screened with a stroke-specific questionnaire and made to undergo a complete neurological examination by a neurologist. Stroke diagnosis was based on clinical evaluation using WHO criteria.

**Results:**

Out of the numbers that were screened, 18 probable stroke cases were identified in the first stage, and of these, 17 fulfilled WHO criteria for stroke, giving a crude prevalence rate of 1.31/1000 population. The prevalence of stroke was higher among the males than the females (1.54/1000 vs. 1.08/1000) with a ratio 1.4: 1. Sixteen subjects (94.1%) had one or more risk factors for stroke. Uncontrolled systemic hypertension (82.4%) was the commonest risk factors for stroke followed by transient ischaemic attack (TIA) (41.2%).

**Conclusion:**

Stroke is a condition that is prevalent in our environment; especially in older adults and men. Uncontrolled systemic hypertension and previous transient ischaemic attacks were the commonest risk factors for stroke in our community.

## Introduction

Stroke is a non-communicable disease with significant socioeconomic consequence worldwide. According to a release by the World Health Organization (WHO) stroke accounts for 10.8% mortality and 3.1% of disease burden worldwide [1]. There is an epidemiologic and demographic transition of diseases in most developing countries with increase risk for cardiovascular diseases [2]. It has been projected that by the year 2030, about 80% of all stroke cases will occur in low and middle income countries of the world [3]. A recent review on global stroke cases showed that while there is a decline in stroke incidence of in developed countries, most developing countries are experiencing a rise in stroke incidence of about 100 percent [4]. Available information on risk factors and epidemiology of stroke are mostly based on western populations, with few data from developing countries, especially Africa [5, 6]. In Nigeria, there are few studies on community estimates of stroke prevalence [7, 8] and many still rely on hospital-based studies which are flawed by methodology since close to one third of stroke patients may not get to hospital due to several reasons some of which may result in high stroke mortality in the community [8]. Having an accurate knowledge of stroke prevalence and associated risk factors from a community-based study is important to develop public health interventions and possible measures at reducing the growing epidemic and burden of stroke. Although, there are few community based studies on stroke in Nigeria, [5, 7] none is from the middle-belt region of the country which consists of 6 states. This therefore necessitated this study carried out in three semi-urban communities of Ilorin emirate council in Kwara state which is in the middle belt of Nigeria and the gate way between the northern and southern part of the country. The study populations consist of mixed agrarian and trading populace.

## Methods

### Study design

This was a descriptive cross sectional study on stroke epidemiology among individuals from the age of 18 years and above in three semi-urban communities in Ilorin, middle-belt region of Nigeria. It was carried out between October 2009 and August 2010.

### Study setting

This study was carried out in Kwara state, which is one of the 36 states in Nigeria and is located in the western part of the middle belt region. It comprises 16 local government areas with Ilorin as the state capital. The population of Kwara State is 1,548,412 based on the 1991 National Census [9]. Literacy level in the state is 50.4% and the people are predominantly farmers with low socio economic status. The state covers a total of 32,500 square kilometers and it has international boundary with Benin Republic along the North-Western part of the state. For the purpose of this study, A semi-urban is one with population of between 500 and 5000 with basic amenities like secondary and primary school, electricity and also has primary health care centre with few private clinics [10]. We randomly selected three local government areas (LGAs) from the 16 local governments in the state namely Ilorin West, Ilorin East and Asa LGAs. In each of these LGAs, one community that met the definition of semi-urban community was selected.

### Participants' selection

Ilorin metropolis and Asa LGAs has eighteen electoral wards using the derivation of wards by Kwara state electoral commission [9, 11]. All the wards have the same degree of urbanization and are socio culturally homogenous. To be included in the study, a participant must have resided in the communities for 6 months and aged 18 years and above at the time of the survey.

### Survey instrument

Our study used the modified version of survey questionnaire developed by the World Health Organization (WHO) protocol for Epidemiological Neurological Disorders in developing countries which has been used by earlier studies [1, 7, 12]. The sensitivity of the screening instrument was 96% for stroke and specificity was 86% [12]. The interviewers were trained by the neurologist in the research team.

### Phase I

At the community level, house to house screening of residents was undertaken by trained interviewers. During the survey, non-residential buildings (e.g. offices, schools) were not surveyed to avoid including persons not normally resident in the LGAs. In addition, demographic and other relevant clinical information were also obtained from the participants or household member such as a parent or first degree relative, if the person was unavailable or unable to respond appropriately to the questions asked. In order not to miss out any case of stroke that was not around during the weekdays, the survey was extended to the weekends and public holidays. For those that travelled out of their locality, two or more visits were re-scheduled when necessary.

### Phase II

Those who were adjudged to be positive for stroke were screened with a stroke-specific questionnaire and made to undergo a complete neurological examination which was performed by a neurologist on the same day of the visit in their homes. The questionnaire included the initial screening questions and additional stroke-specific questions to further validate the diagnosis. We also obtain the duration of stroke, events and symptoms that preceded the stroke, drug history and identified the risk factors for stroke.

### Diagnostic criteria

The burden of stroke was determined as point prevalence, and defined as the proportion of patients who had stroke as at the day of contact with the researchers. We defined stroke according to the WHO criteria as “rapidly developing clinical signs of focal (or global) disturbance of cerebral functions, lasting more than 24 hours or leading to death, with no apparent cause other than that of vascular origin.” [13]. Ischaemic cerebral infarction and intracerebral hemorrhage were included, but transient ischaemic attacks were excluded. As brain computerized tomography (CT) scan was not available in the communities at the time of study; hence, we could not distinguish between cerebral thrombosis, cerebral embolism and intracerebral haemorrhage. The diagnoses were made exclusively on clinical grounds. The diagnosis of stroke was considered definite if (1) Physicians had already diagnosed stroke and study neurologist agreed and (2) study neurologist found presenting sequelae consistent with such a diagnosis.

### Ethical approval

Ethical approval for the survey was obtained from the University of Ilorin Teaching Hospital prior to data collection. Community approval and entry was facilitated after interacting with the heads of these communities and other community leaders and also by meeting with the health workers of each designated community. The purpose of such meetings was to explain the aim of the survey and to obtain communal consent for the study.

## Results

**Figure 1:**
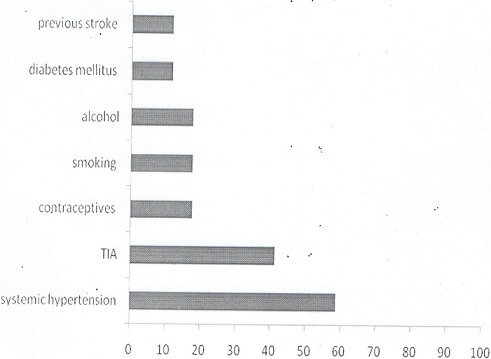
Risk factors for stroke

During the survey a total of 12,992 respondents in the three selected local governments which comprises of Ilorin East, Ilorin West and Asa were recruited into the study. Of these, 6497 were females and 6495 were males. Out of the numbers that were screened, 18 probable stroke cases were identified in the first stage and of these, 17 fulfilled WHO criteria for stroke giving a crude prevalence rate of 131/100,000 population. Out of the identified stroke cases, 10 (58.8%) were males and 8 (41.2%) were females. The male to female ratio was 1.4:1. The mean age of the stroke cases was 58.2± 11.5 years. One (5.9%) of the index stroke case was between the age 30-39 years, three (17.6%) were between the age 40-49 years. Five cases (29.5%) belong to age 50-59 years. While six (35.3%) cases fall within age 60-69 years and one case each (5.9%) fall within ages 70-79 and 80-89 years, respectively (Table 1). The median duration of stroke was 2.5 years with an interquartile range of 1.0-5.0 years and their mean body mass index was 22.3± 5.4kgm-2. The mean systolic and diastolic blood pressures were 150±28mmHg and 90± 20mmHg, respectively (Table 2). The risk factors for stroke identified in the index cases were systemic hypertension (14; 82.4%) followed by transient ischeamic attack (7; 41.2%), while three index cases (17.6%) had history of tobacco smoking, excessive consumption of alcohol beverages and use of hormonal oral contraception, respectively. Two cases (11.8%) had diabetes mellitus which was confirmed on the field by fasting blood glucose measurement and another two (11.8%) had repeat stroke. Five (29.4%) cases had one risk factors and 11 (64.7%) had two or more risk factors for stroke (Figure 1). Among the index cases that were hypertensive, only 7(58.3%) were on antihypertensive medication while none of the diabetics were on glucose lowering agent.

**Table 1:** Demographic and physiologic variables

Variables	Number	Percentages
Male	10	58.8
Females	8	41.2
BMI (kg/m^2^)		
<24.9	11	64.7
25-29.9	3	17.6
≥30	3	17.6
Stroke duration (years)		
1-5	13	76.5
≥6-14	4	23.5
Systolic BP (mmHg)		
≤139	5	29.5
≤140	12	70.5
Diastolic BP (mmHg)		
≤89	7	41.2
≥90	10	58.8

**Table 2:** Age and sex prevalence of stroke

Age category	Total population		Males		Females	
n/1,000	No of strokes/Population size	Stroke Prevalence n/1,000n/1,000	No of stroke/pop size	Stroke Prevalence	no of stokes/pop size	Stroke prevalence
<18	0/1967	0	0/1002	0	0/964	0
20-29	0/3377	0	0/1535	0	0/1842	0
30-39	1/2190	0.46	1/1046	0.96	0/1144	0
40-49	3/1477	2.03	1/784	1.28	2/1144	1.75
50-59	5/837	5.97	4/459	8.71	1/693	1.44
60-69	6/500	12.00	3/272	11.03	5/228	2.19
70-79	1/249	4.01	1/272	3.68	0/113	0
≥80years	1/190	5.26	1/136	7.3	50/91	0
= Overall	17/12,992 1.31		10/6495	1.54	7/6497	1.08

## Discussion

The findings from this study showed that the point prevalence of stroke in semi-urban communities in Ilorin middle belt Nigeria is 134/100,000 with a male predominance. Most of the stroke survivors in this study were within age 60-69 years. The risk factors for stroke identified were: systemic hypertension, previous transient ischeamic attack (TIA), tobacco smoking, and excessive consumption of alcohol and use of hormonal contraception, which is consistent with earlier studies [14]. The world wide prevalence of stroke in all age groups varies between 4 and 20 per 1000 population [2, 4, 14], which is higher than 1.31/1000 found in this study. The prevalence of stroke in this study is higher than 58/100,000 and 114/100,000, reported by two earlier studies among rural and semi-urban populations in western Nigeria, respectively [7, 15]. A more recent community-based study from Niger Delta region of Nigeria reported a crude prevalence of 851/1000 [16]. When compared with result of earlier similar house-hold survey like ours, the prevalence of this study is similar to 143/100,000 reported from rural Kashmir in India [17] but lower than 174/100,000 from rural Bolivia [18]. Much higher values were reported from Bombay India (424/100,000), [19] 461/100,000 in Cotonou, Benin, [20] 620/100,000 in China [21]; 647/100,000 in Cuzco Peru [22] and 595/100,000 in Taiwan [23]. The lowest value was from Papua New Guinea where there was no reported case of stroke among 213 populace [24].

The observed trend of increasing stroke prevalence with age and male preponderance is consistent with findings from earlier studies [4–7, 15, 18]. Plausible reasons for this observation include the fact that the prevalence of systemic hypertension, tobacco smoking and use of alcohol which are leading cardiovascular risk factors tend to be higher in the men. The increasing prevalence of stroke occurred in both sexes and the highest prevalence was found within age 60-69 years. In this study, one (5.9%) case of stroke was found below the age of 40 years which is similar to 2/57 cases found in Egypt [25]. This however is in contrast to 30% of stroke cases reported below the age of 45 years among Pashtun population in Karachi, Pakistan [26]. The leading risk factor for stroke identified in this study was uncontrolled systemic hypertension similar to earlier reports [2, 7, 27]. Only half of the cases that were hypertensive were using antihypertensive medication. Of those on antihypertensive medication, majority (85.7%) had poorly controlled blood pressure with value greater than 140/90mmHg. This trend has been reported in both hospital-based and community cross sectional studies in Nigeria and other parts of the world [28]. The risk of stroke has been strongly related to elevated systolic and diastolic blood pressure [28, 29]; and it appears to have log-linear relationship throughout all ranges of arterial blood pressure among the population [30, 31]. Transient ischeamic attack was the second leading risk factor. In an earlier report one-third of patients with TIA subsequently developed stroke within four years [32].

We found that 17.6% of the stroke cases were obese, smoked tobacco, heavily consumed alcohol and used hormonal contraceptives for family planning. The effect of tobacco smoking has been said to be dose related, as heavy smokers are more likely to develop stroke; however, the risk of having the disease reduces with smoking cessation [32]. Only two (11.8%) of the stroke cases had diabetes mellitus and likewise two (11.8%) of the strokes cases were repeat strokes. The observed low prevalence of stroke in this study may be due to several reasons. These include high stroke mortality in developing countries and rural to urban drift after stroke ictus to seek for better health care. It is also possible that the lower prevalence may be a true reflection of lower stroke rates in our community due to the emerging prevalence of cardiovascular risk factors less than what occurred in the urban cities and westernized countries. The strength of the study is the fact that it is a community-based evaluation and likely to be a true representation of stroke burden in our community; as compared with hospital-based study which depend on patient's health resource utilization. The limitation of the study is the inability to carry our neuroimaging study for the patients due to financial constraint.

## Conclusion

There is a slight increase in stroke prevalence in our community compared to what had been earlier studies. Stroke prevalence increases with age and has a slight male preponderance. Uncontrolled systemic hypertension and previous transient ischaemic attacks were the commonest risk factors for stroke in our community. There is need to educate the community on the risk associated with modifiable risk factors for stroke most especially systemic hypertension.
